# Studies of α-Helicity and Intersegmental Interactions in Voltage-Gated Na^+^ Channels: S2D4

**DOI:** 10.1371/journal.pone.0007674

**Published:** 2009-11-02

**Authors:** Zhongming Ma, Jun Kong, Roland G. Kallen

**Affiliations:** 1 Department of Biochemistry and Biophysics, University of Pennsylvania School of Medicine, Philadelphia, Pennsylvania, United States of America; 2 Mahoney Institute for Neuroscience, University of Pennsylvania School of Medicine, Philadelphia, Pennsylvania, United States of America; Yale School of Medicine, United States of America

## Abstract

Much data, including crystallographic, support structural models of sodium and potassium channels consisting of **S1–S4** transmembrane segments (the “voltage-sensing domain”) clustered around a central pore-forming region (**S5–S6** segments and the intervening loop). Voltage gated sodium channels have four non-identical domains which differentiates them from the homotetrameric potassium channels that form the basis for current structural models. Since potassium and sodium channels also exhibit many different functional characteristics and the fourth domain (**D4**) of sodium channels differs in function from other domains (**D1–D3**), we have explored its structure in order to determine whether segments in **D4** of sodium channels differ significantly from that determined for potassium channels. We have probed the secondary and tertiary structure and the role of the individual amino acid residues of the **S2D4**) of Na_v_1.4 by employing cysteine-scanning mutagenesis (with tryptophan and glutamine substituted for native cysteine). A Fourier transform power spectrum of perturbations in free energy of steady-state inactivation gating (using midpoint potentials and slopes of Boltzmann equation fits of channel availability, h_∞_-V plots) indicates a substantial amount of α**-**helical structure in **S2D4** (peak at 106°, α**-**Periodicity Index (α**-**PI) of 3.10), This conclusion is supported by α**-**PI values of 3.28 and 2.84 for the perturbations in rate constants of entry into (β) and exit from (α) fast inactivation at 0 mV for mutant channels relative to WT channels assuming a simple two-state model for transition from the open to inactivated state. The results of cysteine substitution at the two most sensitive sites of the **S2D4** α**-**helix (N1382 and E1392C) support the existence of electrostatic network interactions between **S2** and other transmembrane segments within Na_v_1.4**D4** similar to but not identical to those proposed for K^+^ channels.

## Introduction

Voltage-dependent ion channels respond to changes in the membrane potential by conformation changes that permit or impede ionic currents [1]. Despite functional similarities within the voltage-gated channel family, substantial differences exist among the behaviors of sodium, potassium and calcium channel subfamilies [1]. It is unclear which structural differences account for these divergent actions despite the fact that it is thought that all of these proteins have similar overall structures. Each is thought to be composed of four subunits surrounding a central aqueous pore that provides the ion permeation pathway. Each subunit contains six transmembrane segments, **S1–S6**, with the first four, **S1–S4**, comprising the voltage-sensor domain (V-Domain), and the other two segments (**S5** and **S6**) creating the loosely associated pore (P-domain) [2]. In most cases a number of conserved arginine residues along **S4** are predominantly responsible for the gating charge controlling the voltage-dependent conformational changes that lead to a concerted pore-opening transition of the channel from the resting state and its subsequent progression to a closed, inactivated state [3], [4], [5], [6], [7], [8], [9].

Understanding voltage-gated channel structure and function requires structural information and much of that to date has been provided by the X-ray diffraction studies of prokaryotic and eukaryotic potassium channels [10], [11], [12], [13], [14], [15]. The current model posits that within each subunit the V-domain contains a highly cationic voltage-sensor paddle (V-paddle, composed of a helix-turn-helix structure [the **S4** segment in an antiparallel relationship to **S3b**, the C-terminal half of the **S3** segment]). It has been suggested that: (i) voltage-gating domains have a large amount of internal flexibility compared with the pore domains, (ii) the V-paddle is situated on the periphery of the V-domain with some cationic sidechains interacting with anionic elements of adjacent subunits **S1**, **S2** and **S3**; (iii) **S2** and **S3** are situated more proximal than V-paddle to the P-domain, and (iv) some **S4** arginine residues interact in a critical fashion with the lipid membrane [2], [4], [16], [17], [18], [19], [20]. Whether a given sidechain is exposed to the internal or external compartment depends upon voltage in a manner that can account for the bulk of gating currents and voltage-dependent sidechain exposure of potassium channels [21]. However, there is less information on the position and localized movements associated with gating for the other segments comprising the V-domain (*c.f.*, [22], [23], [24], [25], [26], [27]). Other K^+^ and Na^+^ channels transmembrane and linker segments have been implicated in voltage sensing, especially **S2** and **S3** [4], [18], [19], [24], [28], [29]. Currently neither the differences in function between potassium and sodium channels (see below) nor the divergent behavior between fourth domain (**D4**) and **D1–D3** of sodium channels are understood in terms of channel structural variations.

**Figure 1:**
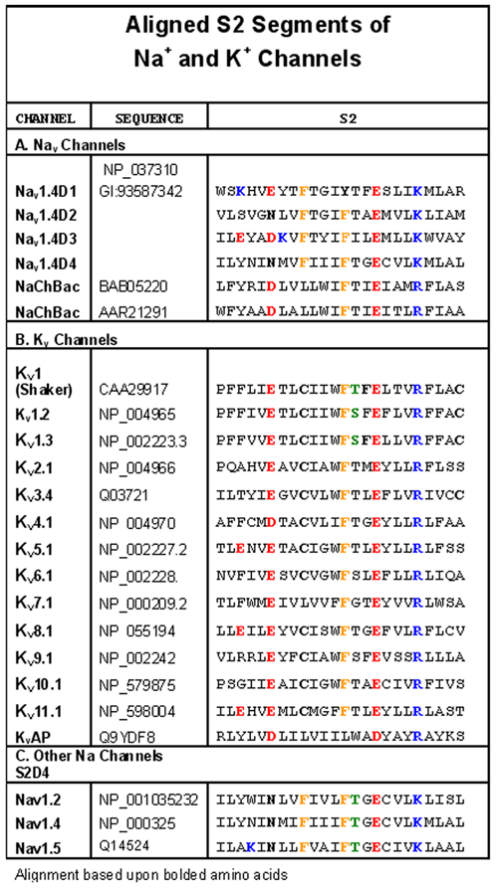
Alignment of the S2 segments of Na^+^ and K^+^ channels.

**S2** segments from many families of eukaryotic and prokaryotic K^+^ channels have in common: (i) two acidic amino acids separated by nine residues (the first and the second are referred to as upstream and downstream, respectively), (ii) an aromatic (phenylalanine or tyrosine) sidechain three residues upstream from the second (downstream) acidic residue in eukaryotic channels, and (iii) a basic residue four residues downstream from the second acidic residue (Fig. 1). Because Na^+^ channels are not tetramers, in contrast to K^+^ channels, the **S2** sequences have evolved to vary significantly from domain to domain (Fig. 1). Especially noteworthy is the change at the conserved upstream acidic residue in K^+^ channels from glutamate or aspartate to a neutral residue, glutamine, in **D2** and **D4** of Na_v_1.4 (Fig. 1) such that the number of **S2** segment anionic residues is one fewer in these domains.

It remains unclear whether these structural differences are responsible for functional divergences between potassium and sodium channels, for example those that contribute to their dissimilar roles in generating action potentials and accomplishing repolarization in the heart as well as in process of inactivation [30], [31]. We undertook experiments using scanning mutagenesis of the **S2** segment of domain 4 of Na_v_1.4 in order to understand the role of each residue in channel function, particularly with respect to any differences between homomeric K^+^ and both homomeric (NaChBac) and non-homomeric Na^+^ channels such as Na_v_1.2, Na_v_1.4 and Na_v_1.5. Because structural similarities do not necessarily predict function further experiments of S2 segments seemed warranted, especially those that include non-diffraction data [32].

We have addressed two questions in this communication: Is the **S2D4** transmembrane segment α-helical as proposed for **S2** segments in potassium channels? What are the consequences in terms of function of mutating **S2D4** residues, especially at the “high impact” residues [28] postulated to be involved in interactions with sidechains on other transmembrane segments, notably the voltage-sensing **S4** segment?

The rationale for evaluating the secondary structure and solvent exposure of **S2D4** and several **S2–S3** loop amino acids using cysteine-scanning mutagenesis is that an α-helical transmembrane segment has two faces that may contribute qualitatively or quantitatively different effects upon channel behavior. Substitution with cysteine changes the sidechain characteristics and also enables study of intra- and extracellular solvent accessibility with membrane-impermeable methanethiosulfonate (MTS) reagents *via* the substituted cysteine accessibility method (SCAM) [33], [34], [35], [36], [37]. The mutation-induced alterations in channel electrophysiological properties (gating and kinetics of inactivation) are consistent with a major portion of **S2D4** being α-helical, similar to the situation with K^+^ channels [28], [38]. The study reported herein also revealed important interaction sites on **S2D4** supporting their probable involvement with cationic sites elsewhere in the channel within charge networks, again similar to those apparently present in K^+^ channels [39]. This study, however, finds no evidence for structural specialization of S2D4 despite the known functional differences cited earlier.

## Results

### Perturbation of Channel Gating by S2D4 Cysteine-Scanning Mutations

We tested the presumption that **S2D4** is an α-helix with two faces exhibiting different sensitivities to amino acid substitution by analyzing cysteine-scanning mutations (with tryptophan and glutamine substituted for native cysteine) of the rNa_v_1.4 **S2D**4 segment (residues 1377 to 1400) and several **S2–S3** loop flanking amino acids (1401, 1405, 1409) expressed in the tsA20l cell line by patch-clamp methods. Because all mutant genes produced functional channels, as expected for usually well-tolerated cysteine mutations, we have a complete data set.

**Figure 2:**
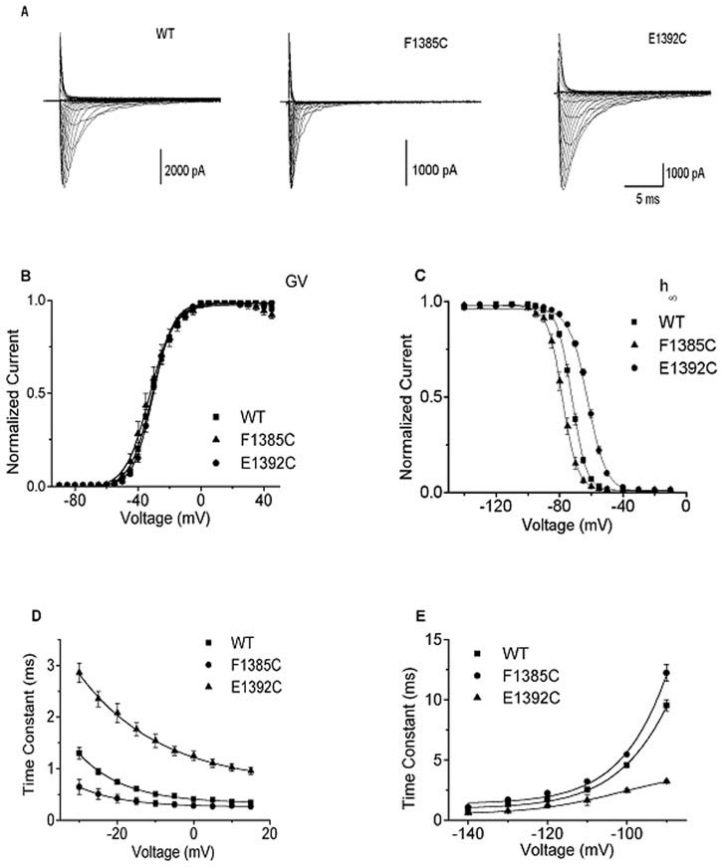
Extremes of gating phenotypes of cysteine-substituted S2D4 mutants, F1385C and E1392C compared with wild-type channels. **A**, Family of currents recorded from tsA201 cells expressing WT (left) and the most positively (F1385C, middle) and negatively perturbed mutant channels (E1392C, right) in terms of h_∞_ (see 1C below). Current families were elicited by voltage pulses from a holding potential of −120 mV rendered in 5 mV increments. **B**, Current-voltage (G–V) relations for WT and mutant channels (E1392C, F1385C) in A. **C**, Availability-voltage (h_∞_-V) relations for WT, F1385C (largest hyperpolarizing shift) and E1392C, (largest depolarizing shift). Symbols are normalized (I/I_max_) values and solid lines are single Boltzmann fits to the data (most significant parameter perturbations contained in Table 1 the values for the entire set of mutant channels are contained in the Table A, Appendix S1)). **D**, Current decay time constants (τ_h_). **E**, Recovery from inactivation time constants (τ_rec_). The values for τ_h_ and τ_rec_ for the entire set of mutant channels are contained in the Table B Appendix S1.

The electrophysiological examination included comparison of the families of currents, G–V and h_∞_-V curves, and the voltage-dependence of time constants for entry into and recovery from fast inactivated states using wild-type and mutant channels. Examples of families of currents for WT channels and mutations at two positions that were among the most sensitive to cysteine substitution (1385 and 1392) in terms of h_∞_ shifts are shown in Fig. 2A.

Clearly F1385C and E1392C channels exhibit more rapid and less rapid current decay kinetics, respectively, compared with WT Na^+^ channels. The changes in steady-state activation parameters were relatively small (G–V, Fig. 2B, Table 1 and Appendix S1, Table A). This failure to see substantial effects on activation by mutations in **D4** was not unexpected since Na^+^ channels appear to have differentiated their domains such that **D4** is substantially more involved with inactivation than with activation [3], [40]. Consistent with this view, in contrast to the minor changes in activation, there were significant changes in inactivation at six sites of **S2D4** (>1 kcal/mole: N1382C, I1386C, F1389C, T1390C, E1392C, K1396C) as seen in steady-state availability-voltage (h_∞_-V) relationships (Fig. 2C and Appendix S1, Table B). The two largest shifts in midpoints E1392C (rightward) and F1385C (leftward) relative to WT indicate destabilization and stabilization of the inactivated state relative to non-inactivated states, respectively. The right shift in the h_∞_-V plot for E1392C is primarily due to the combined effects of a slower rate of entry into the inactivated state and a more rapid rate of recovery from inactivation while the left shift for F1385 is the result of a more rapid rate of entry into and slower rate of exit from the inactivated state (Fig. 2D and 2E, Table 2 and Appendix S1, Table B).

**Table 1:** Gating Properties (G–V) of WT and S2D4 Segment Mutants of Na_v_1.4.

Channel	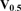 (mV)	z (e_0_)	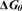 (kcal/mol)	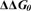 (kcal/mol)
WT (11)	−31.4±1.04	4.04±0.10	−2.94±0.17	
N1382C (5)	−30.5±1.17	4.36±0.17	−3.10±0.19	−0.15±0.21
F1385C (6)	−33.2±2.24	3.84±0.17	−2.93±0.31	−0.02±0.33
I1386C (4)	−33.9±1.11	4.26±0.15	−3.34±0.21	−0.39±0.27
F1389C (4)	−35.4±1.32	4.22±0.29	−3.45±0.32	−0.51±0.35
E1392C (15)	−29.6±1.21	4.35±0.19	−2.99±0.23	−0.05±0.26
C1393Q (5)	−30.9±1.55	4.11±0.16	−2.92±0.16	0.02±0.23

**Table 2:** Gating Properties (h_∞_-V) of WT and S2D4 Segment Mutants of Na_v_1.4.

Channel	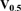 (mV)	z (e0)	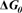 (kcal/mol)	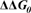 (kcal/mol)	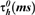	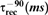
WT (11)	−72.2±0.58	5.85±0.10	−9.77±0.18		0.41±0.01	9.52±0.47
N1382C (5)	−62.4±1.09**	4.58±0.34**	−6.74±0.42**	3.03±0.46	0.64±0.07*	4.96±0.08**
F1385C (6)	−78.2±0.92**	5.55±0.12	−10.01±0.20	−0.23±0.27	0.28±0.04*	12.2±0.71*
I1386C (4)	−65.9±0.57**	5.27±0.05**	−8.01±0.07**	1.77±0.19	0.67±0.03**	2.67±0.43**
F1389C (4)	−69.3±0.90*	4.79±0.18**	−7.67±0.30**	2.11±0.34	0.63±0.04**	5.48±0.74**
E1392C (7)	−62.0±1.05**	4.65±0.14**	−6.70±0.28**	3.09±0.33	1.24±0.09**	3.21±0.13**
C1393Q (5)	−70.1±1.09	5.67±0.29	−9.13±0.33	0.64±0.37	0.55±0.05	10.7±1.15**

**Figure 3:**
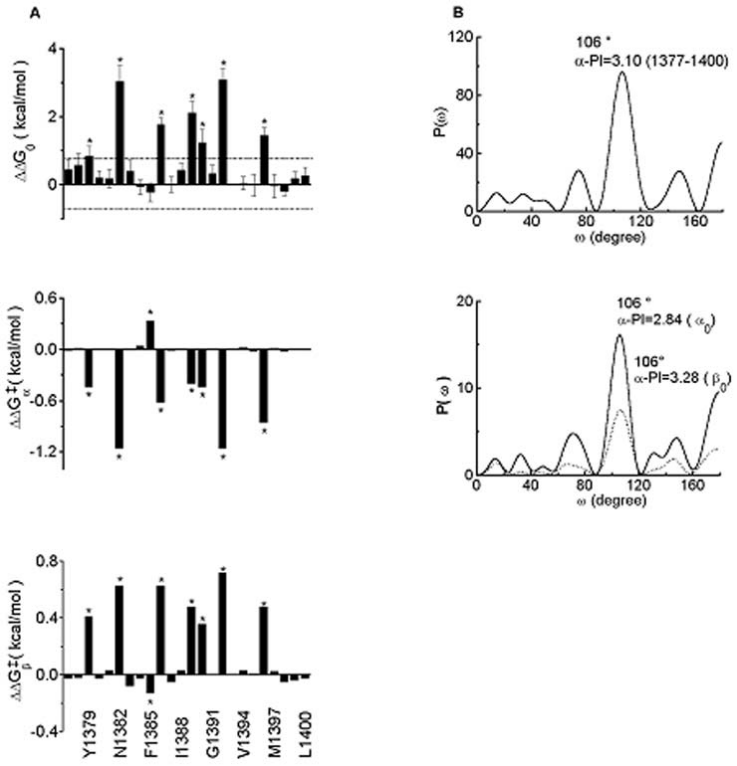
Periodicity in the distribution of free energy perturbations of inactivation gating at serial cysteine substitutions in S2D4 as a function of residue position (numbers) in the primary sequence. **A**, Steady-state inactivation (upper); time constants for exit (middle), and for entry (bottom) from and into inactivated states. **B**, Fourier transforms for data for steady-state inactivation (top) and for rate constants for exit and entry from and into inactivated states (bottom).

The evaluation of effects of mutations on inactivation relied upon ΔΔG_o_ values calculated from the midpoint (*V_0.5_*) and slope (−*RT/zF*) factors (Materials and Methods). Note that the midpoint and slope contributions may have opposing effects such that a large leftward shift in *V_0.5_* may be offset by an increase in slope and as a result the observed free energy change for steady-state inactivation may be minimized (*e.g.*, F1385C, Table 2). A plot of the ΔΔG_oi_ values for steady-state inactivation for all the mutations reveals mainly positive differences consistent with amino acid (mostly cysteine) substitutions causing destabilization of the non-inactivated states relative to inactivated states (Fig. 3A, upper).

**Figure 4:**
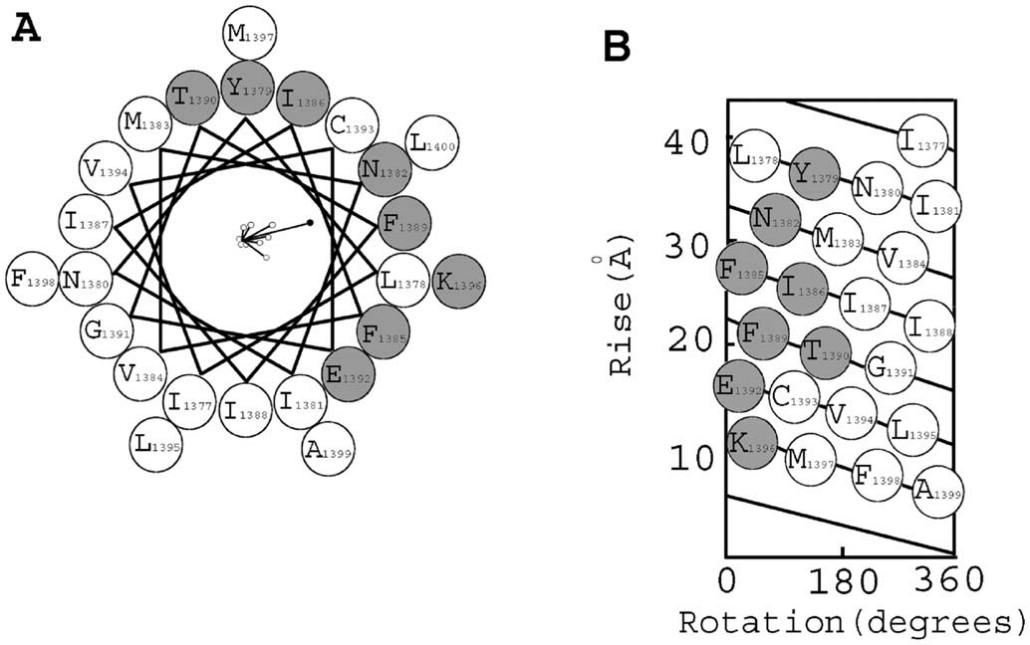
Helical wheel and net representations of free energy perturbations of steady-state inactivation in S2D4. **A**, Helical wheel representation of magnitude of perturbations *ΔΔG_oi_* induced by substitution (mostly cysteine) for the 24 amino acid segment (1377 to 1400) (the individual vectors (open circles) and the vector sum (closed circle) are at the center of the helix viewed from the extracellular surface of the channel). **B**, Helical net representation localizing perturbations to one side of the helix.

The peaks for the ΔΔG_oi_ *vs.* site for steady-state inactivation for residues 1377–1400 appear to be consistently separated by two or three residues, a pattern that indicates α-helical periodicity in the data for the **S2D4** segment, a point that is more obvious from the primary peak at 106° in the Fourier transform power spectra (Materials and Methods) (Fig. 3B, upper and 3B, lower). The calculated α-PI value for **S2D4** (residues 1377–1400) of 3.10 is consistent with the conclusion that this transmembrane segment is substantially α-helical in conformation (Fig. 4) [41]. Note that helices imbedded within plasma membranes tend to show peaks shifted to slightly higher values than the 100° predicted for an α-helix [41].

The large vector sum based on the free energy perturbations of steady-state inactivation shown in the helical wheel clearly shows the localization of perturbations to one side of a helix (Fig. 4) [28], [38]. Thus, fully 50% of the residues (low impact) show no perturbation by substitution, perhaps because they are associated with plasma membrane lipids. The sidechains in this group are mainly hydrophobic and branched chain amino acids (Met, Leu, Ile, Val) or those with small sidechains (Gly, Ala, Cys). The single exception is Phe^1398^ for which we have not found a phenotypic difference thus far possibly because it is too distant from the center of the membrane or from protein-protein interactions. The amino acids on the substitution-sensitive face of the helix in general have functional groups with additional properties: charged, aromatic, hydrogen bond donors and acceptors, *etc*. (Glu, Lys, Tyr, Phe, Asn, Thr, although there remains the exception of Ile^1386^).

### Inactivation Kinetics

Since steady-state inactivation in a normally coupled Na^+^ channel reflects the rate of entry and departure from the fast inactivated state, shifts in the midpoint of h_∞_ will be reflected in the τ_h_ and/or τ_rec_ values. Time constants for current decay and recovery from inactivation as a function of voltage were, in a large number of cases, indistinguishable from those of WT channels. Significant changes in τ_h_ values at 0 mV and in τ_rec_ values at −90 mV were found at residues 1379, 1382, 1385, 1386, 1389, 1390, 1392, 1396 (Appendix S1, Table B). The kinetic data seem to be more sensitive to substitution than the steady-state h_∞_ data perhaps because the h_∞_-V midpoint potential can experience compensatory changes for entry into and exit from the inactivated state as noted above. Rate constants for entry into (β) and exit from (α) the inactivated state at 0 mV, assuming a simple two state (*Non-inactivated*↔*Inactivated*) model, were obtained from fits to the τ_h_ and τ_rec_ values over the voltage-range studied as described in the Experimental Section a subset of which are collected in Table 3 (see Appendix S1 Table C for entire data set). A plot of the ΔΔG_oi_^‡^_(entry)_ and ΔΔG_oi_^‡^_(exit)_ values of the mutant channels as a function of position shows a periodicity of “high impact” residues [28] (Fig. 3A, middle and lower panels) corresponding approximately to that of ΔΔG_oi_ values for steady-state inactivation (Fig. 3A, upper). The power spectrum analysis, which spans the entire **S2D4** segment, provides α-PI values of 3.28 and 2.84 with peaks at 106° and 106° for entry and exit, respectively, consistent with the designation of this entire segment as largely an α-helix (Fig. 3B, lower). When these data from kinetics of entry and exit from the inactivated state are plotted on helical wheel and net representations (not shown), the high impact residues congregate on one surface of the α-helix as shown earlier for steady-state inactivation (Fig. 4).

**Table 3:** The Rate Constants of WT and S2D4 Segment Mutants of Na_v_1.4 for Entry Into (β) and Exit From (α) the Fast Inactivated State.

	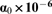 (1/ms)	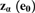	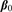 (1/ms)	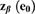	ΔG_α0_^‡^ (kcal/mol)	ΔG_β0_^‡^. (kcal/mol)	ΔΔG_α0_^‡^ (kcal/mol)	ΔΔG_β0_^‡^. (kcal/mol)
WT (11)	3.50	2.80	10.0	2.25	20.93	12.07		
N1382C (5)	25.0	2.45	3.50	2.35	19.75	12.69	−1.17	0.63
F1385C (6)	2.00	2.80	12.5	2.25	20.26	11.94	0.33	−0.13
I1386C (4)	10.0	2.85	3.50	2.25	20.30	12.69	−0.63	0.63
F1389C (4)	7.00	2.70	4.50	2.25	20.51	12.54	−0.41	0.48
E1392C (15)	20.0	2.40	2.00	2.25	19.75	12.79	−1.17	0.72
C1393 Q(5)	3.65	2.65	9.50	2.65	20.90	12.10	−0.03	0.03

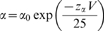
 and 
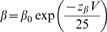
, V is the membrane potential in mV, 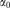 and 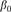 are the rate constants at 0 mV, 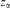 and 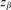 are equivalent charge in the gating process (see text) and 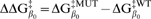 or 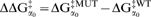.

### Effects of MTSET Exposure

We analyzed whether changes in electrophysiological properties (currents, G–V, h_∞_-V, τ_h_ and τ_rec_ values) occurred upon exposure to MTSET for each of the cysteine-scanning mutations. We took precautions to avoid reaction of MTS reagents by their passage through the membrane or by virtue of some leakage of reagent as the microelectrodes formed the patch prior to the establishment of the whole cell recording configuration. Thus, we routinely used: (i) a charged MTS reagent (MTSET) thought to unable to traverse the plasma membrane, (ii) a scavenger thiol (L-cysteine) on the contralateral side, and (iii) for cytoplasmic-presented reagent, MTS-containing microelectrodes were backfilled with reagent-free solution to avoid reagent leakage that might produce artifacts due to reaction with extracellular surface residues during establishment of the seal.

For externally applied MTSET the only changes that might be significant in activation parameters (G–V), are at positions 1378 and 1382 (Appendix S1 Table A). For internally applied MTSET there are no significant changes in any parameters (data not shown). The caveat remains that sites may react with MTSET and yet not show a change in channel characteristics.

### S2–S3 Segment Mutations

A screen of intracellular residues adjacent to S2 in the four domains included the following mutations: **D1**, T187C/F188Q **D2**, P628A, Y629Q, Y631(Q,C,F,T,E,K,N,R),Q633A **D3**, Y1085Q, F1086Q **D4**, R1401(Q,C), H1402(Q,C), Y1403(Q,C), Y1404(Q,C), F1405(Q,C), T1406(Q,C), I1407(Q,C) G1408C, W1409C. No significant perturbations of steady-state activation or inactivation midpoints or slopes were seen (data not shown).

## Discussion

**Figure 5:**
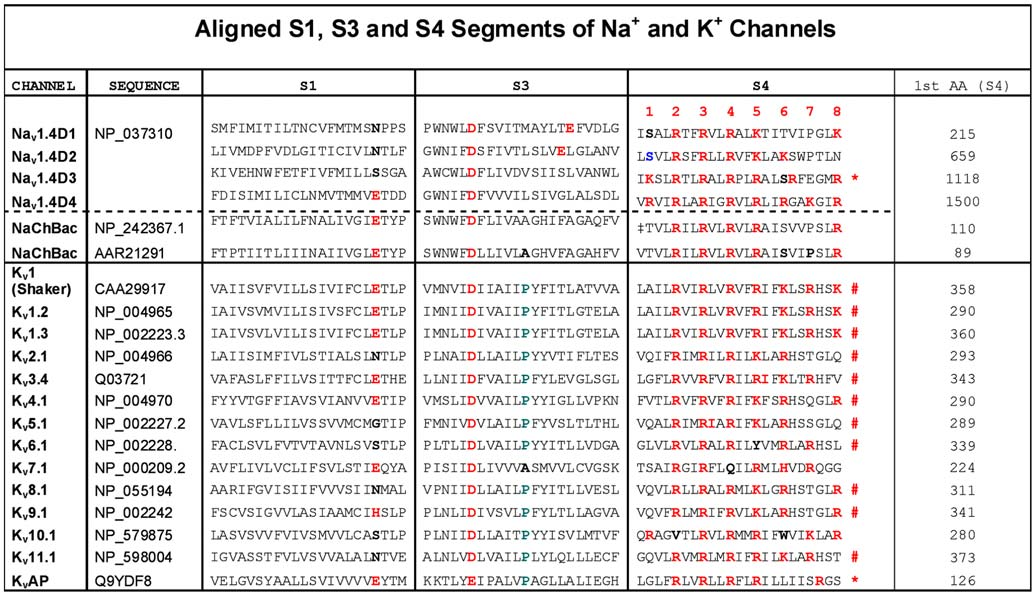
Alignment of S1, S3 and S4 segments in Na^+^ and K^+^ channels. This alignment of NaChBac exhibits greater identity in S1 with S1D1 than that published previously (7 of 22 identical (*c.f.*, 0 of 22 residues) [39]. The positions of the cationic sites in S4 are numbered 1–8. *, cationic site offset one residue; #, doubled cationic sites (RH), see text. The numbering system used here causes the designation of K^+^ channel cationic residues to differ from that elsewhere in the literature: 2nd position here is R1 of Shaker [4]. R0 in K_v_2.1 need not be invoked with this alignment (*c.f.*, [2]. ‡ S3 and S4 appear to overlap by one residue.

Many transmembrane segments of integral membrane proteins are composed of amphipathic *α* **-**helices with one face interacting with lipid and the other with protein and containing a hydrophobic core. Prominent examples are the photoreaction center and cytochromes, in which the hydrophobic core favors electron transport [13], [14], [39], [41], [42]. We instituted an investigation of the structural and functional roles of residues of Na^+^ channel transmembrane segments for the following reasons: (i) ion channels contain an aqueous pore accommodating ion transport making them quite different than electron transport proteins [43]; (ii) there are obvious primary sequence differences among transmembrane segments of various ion channels (see Na^+^ and K^+^ channels, Figs. 1 and 5); (iii) functional specialization exists among Na^+^ domains [3]; and (iv) there is functional variation among individual residues even among V-domains of K^+^ channels [32]. On these grounds we initiated an investigation of the structural and functional consequences of serial cysteine-substitution and SCAM in Na_v_1.4 beginning with **S2D4** [37]. This study explored the secondary and tertiary structure of this transmembrane segment, the functional effects of substitution at each residue, and exposure of sidechains to extracellular and intracellular compartments.

### Voltage-Sensing Domain

***S4*** *.* For the Shaker (K_v_1) and Shab (K_v_2.1) channels as well as Na_v_1.4, opening is coupled to displacement of 3–4 charge units/voltage sensor across the membrane-voltage difference [4], [5], [44], [45], [46], [47]. In Shaker, the arginines R2, R3, R4, R5, each contribute approximately a single charge and K6 contributes approximately half a charge [4], [5] (numbering of Fig. 5, Table 4): this is referred to by us as a “2–6 S4 cationic sidechain motif” [4], [5]. The crystal structure of the open channel conformation is consistent with these electrical measurements if the cationic sidechains 2–5 move from inside to outside and that at site 6 moves from a midway point to outside upon channel opening (‘outside’ and ‘inside’ refer to extra- and intracellular compartments). In Na_v_1.4**S4D4** sites 2–6 are 1451, 1454, 1457, 1460, and 1463. The occurrence in Na_v_1.4 **S4D4** of eight cationic sidechains compared with seven in many other **S4** segments, especially in K_v_ channels, led to our assignment of the outermost cationic site in the latter **S4** segments as site number 2 (*c.f.*, [48]) (Fig. 5). Thus, the earlier designations of R0(294), R1(297), R2(300), R3(303), R4(306), K5(309), R6(312) refer to S4 cationic sites 2–8 in the present nomenclature: It has been suggested that a shorter **S4** exists with site 8 assigned to the 4–5 linker [48].

**Table 4:** Numbering System Employed.

	Shaker	K_v_1.2	NaChBac*	rNa_v_1.4D4
S1 (E)	217	183	43	1366
S2 (E)	283	226	60	1382(N)
S2 (E)	293	236	70(D)	1392
S2 (R)	297	240	74	1396(K)
S3 (D)	316	259	93	1413
S4 (+)				
1	359	291	110	1441
2	362	294	113	1444
3	365	297	116	1447
4	368	300	119	1450
5	371	303	122	1453
6	374	306	125	1456
7	377	309	128	1459
8	380	312	131	1462

Italicized, bold, underlined sites have no cationic sidechain; alternative residues in parentheses.

### Intersegmental Electrostatic Interactions Among Residues of the V-domains of K^+^ Channels

Crystallographic data has confirmed that the transmembrane segments of K^+^ channels are predominantly α-helical [13], [28], [38], [48]. Our focus is upon the electrostatic network interactions among transmembrane segments of the V-domains involving highly conserved acidic groups on **S2** and **S3** and basic residues on **S4**. This conservation is revealed in alignments of amino acids in **S1–S4** segments based upon the spacing of the transmembrane segments and the occurrence of conserved amino acids (**S1**, Glu and Pro; **S2**, two Glu/Asp sites, **S3**, Asp/Glu and Pro; and **S4**, constellation of cationic residues) (Figs. 1 and 5).

**Figure 6:**
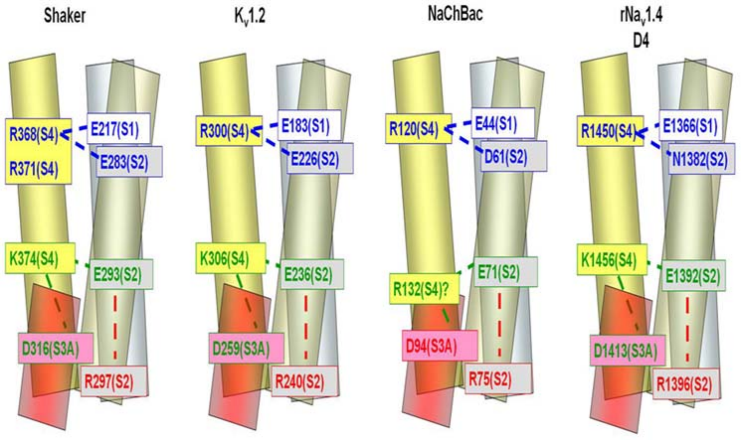
Models of residue interactions in V-domains K_V_1.2. NaChBAC and Na_V_1.4. S1(blue), S2 (gray), S3a (pink) and S4 (yellow). For an explanation of the question mark at D129 in NaChBac see text and note that numbering now corresponds to that of AAR21291 (*c.f.*,[39]).

In the Shaker K^+^ channel these sidechain interactions were originally revealed by their involvement in the surface expression of channels presumably because they stabilize the structure of the K^+^ channel during the maturation of the protein and transit to the surface [4], [18]. The two networks in Shaker are (see Table 4 for numbering systems): an external cluster, E283(**S2**)∶R368(**S4**) (and likely R371(**S4**)) (*i.e.*, **S4** cationic sites 4 and 5) and an internal cluster, E293(**S2**)∶D316(**S3**)∶K374(**S4**) (*i.e.*, **S4** cationic site 6, Fig. 6). The involvement of an **S1** anionic site illustrated for Shaker was not apparent in the earlier studies and is inferred from the crystallographic structure of K_v_1.2 [48]. The comparable V-domain networks for K_v_1.2 are 15 Å apart: external network E183(**S1**)∶E226(**S2**)∶R300(**S4R4**)∶R303(**S4R5**) resides in an external aqueous cleft and internal network E154(**S0**)∶E236(**S2**)∶D259(**S3**)∶K306(**S4K6**)∶R309(**S4R7**) appears not to be exposed to aqueous solution (Table 4 and Fig. 6) [48]. The functional importance of **S4R7** is, however, unclear since many S4 segments do not have a cationic residue at this position (Fig. 5). The intrachain interactions of Glu with Arg at the cytoplasmic end of **S2** derive from modeling of NaChBac (Fig. 6) [39]. Evidence supporting the functional importance of electrostatic interactions in the Shaker channel during gating has been provided by charge-reversal mutations at 283 and 371, which stabilize an activated conformation, whereas charge-reversal at 283 and 368 stabilizes a closed, partially activated (intermediate) conformation [49].

It may be the case that exceptions provide additional support for the postulated “2–6 S4 cationic sidechain motif”. Note that the electrostatic networks are perturbed in K_v_6.1 and 10.1 (also called K_v_6.3) due to the lack a cationic sidechain at **S4** sites 5 or 6 due to the presence of Tyr or Try, respectively, which may account, at least in part, for the fact that these channels are “silent” when expressed alone. When expressed as heterotetramers with, for example, K_v_2.1, the “silent” subunits become modifiers of channel function [50], [51], [52]. Another exception is K_v_7.1 which is missing one cationic group (either site 3 or 4 depending upon the alignment). This may account for its lesser gating charge and that it functions as a leak channel when associated with ancillary subunits such as MiRP2. These deviations may represent specializations among eukaryotic voltage-gated cation channels and account at least in part for the unique loss of voltage dependence of KCNQ1 as well as its conversion from a voltage-dependent, slowly activating I_K_s repolarization current to a potassium ion leak conductance when associated with auxiliary subunits channels [53].

In sum, the V-domains of almost all functional K^+^ channels possess conserved charged residues that are available to interact *via* electrostatic interactions (Fig. 6) [54].

### The Secondary Structure of the S2D4 Segment in rNa_v_1.4 (Residues 1377 to 1400) is α-Helical

In the absence of high resolution structures of Na^+^ channels we have turned for structural information to a Fourier transform power spectral analysis of the energetics and kinetics of steady-state inactivation from scanning mutagenesis. This study reveals a periodicity consistent with a substantial α**-**helical character within the **S2D4** segment in agreement with revelations from crystallographic studies of K^+^ channels [10], [28], [38]. Such periodicity need not have been present since the residues on one face of the helix would not be uniform with respect to access, environment or function and because states, such as the resting state, are likely comprised of ensembles of rapidly interconverting conformations such that minor changes in interactions are undoubtedly occurring continuously [49], [55]. Nevertheless, we observe periodicity that we attribute to hydrophilic/hydrophobic sidedness or to one face remaining in static contact with other parts of the protein while the opposite face experiences changing contacts (*e.g.*, related to motions that the **S4** segment experiences). Noteworthy is the observation that the two sites that are among the most sensitive to substitution are 1382 and 1392 (Fig. 4), which are positions homologous to the anionic side chains of Shaker **S2** (283 and 293) that are the postulated interaction sites with other transmembrane segments (see below and Table 4).

### Conserved Residues in the V-Domains of Na^+^ Channels

What is the situation with respect to tetradomain Na^+^ channels regarding candidate electrostatic network residues? The varying number of cationic charges in **S4** segments of the various domains of rNa_v_1.4, from five to eight (Fig. 5) might not interfere with residue interactions if the 2^nd^, 3^rd^, 4^th^, 5^th^, and, to a lesser extent, 6^th^ cationic sites are those primarily involved in gating since these are the most conserved positions. The proposed alignment is consistent with the conclusion that the N-terminal part of the **S4** segment underlies gating charge [5], [26], [56]. In addition, this alignment of the **S4** segments allows ion-pairing networks in **S2D4** Na_v_1.4 similar to those proposed to be operative in Shaker and maximizes identities or homologies among the sequences (Table 4, Figs. 5 and 6). Thus, the following charged side chain residues that contribute to electrostatic networks in K^+^ channels are conserved in all domains of Na_v_1.4: **S2**, the inner cluster anionic site (Glu) (and, interestingly, the cationic residue (Lys/Arg) four amino acids farther toward the interior as well as an aromatic amino acid Phe/Tyr three residues N-ter to the inner network Glu); **S3**, the inner cluster Asp at position 6 of the transmembrane helix; and **S4**, cationic residues at positions 2, 3, 4, and 5 (Figs. 5 and 6).

The primary sequence *differences* between Na_v_ and K_v_ channels can be summarized as follows in Na_v_1.4: (i) **S1** segments in **D1** (Asn152), **D2** (Asn585) and **D3** (Ser1037) are *lacking* the negatively charged residue (fourth from exterior surface, which is also true for almost half of the K_v_ channel families in which replacements are Asn, Ser, His and Gly) (Fig. 5, bold black letters); (ii) Asn replacement in **S2** segments of **D2** (N608) and **D4** (N1382) of Na_v_1.4 (see below); (iii) **S3** highly conserved Pro is not present in Na^+^ channels being replaced by Ala, Leu, Val or Met (suggesting that the V-paddle, if it exists in domains of the sodium channel, will be structurally different than that of K_v_1.2); and (iv) **S4**, a shift of the cationic residue one position to the right at the 6^th^ position in **D3** compared with the other domains of Na_v_1.4 and most K_v_ channels (exception K_v_AP) (* Fig. 5).

### Local Network Interactions Sites in Na_v_ Channels. Functional Studies of NaChBac

Studies of the functional effects of substitutions of native acidic residues with alternative sidechains {other negative (Asp/Glu), neutral (cysteine), or positive (lysine)} indicated shifts in G–V midpoint potentials, slope factors and kinetics of activation and inactivation [39]. Combining these observations with molecular modeling led this group to the conclusion that the main interactions in NaChBac are: (1) external network consisting of D60(**S2**), A99G100(**S3**) and R122(**S4R5**); (2) internal network contributed by D70(**S2**), R131 (**S4R8**) and D93(**S3**) with R74(**S2**); and (3) an intrasegmental helix-stabilizing interaction between D70 and R74 of **S2**: the latter may account for the high conservation of. Arg/Lys at the cytoplasmic end of **S2** (Figs. 1 and 6) [39]. Note, first, that our numbering is based on NP_242367.1 and differs by 2 from that given in reference [39] and, second, that the postulated interactions involving the external and internal negative clusters do not correspond to those in the K^+^ channels upon which the model is based. In NaChBac: the internal interaction in the model is with **S4** site 8 (not with 6 and 7 as is generally the case) and this discrepancy is largely the result of absence of cationic sidechains at sites 6 and 7 (Fig. 5). The dramatic difference in the internal negative cluster interactions results from pairing D70 with a sidechain almost two complete helical turns further toward the cytoplasmic end of S4 (accounting for the question mark at R131 in Fig. 6) [39]. It may be the case that in the absence of cationic sidechains at 125 and 128, the calculations that underlay the proposed E70(**S2**)***-***R131 interaction may have been biased toward the involvement of R131. Without additional experimental evidence, it is difficult to further evaluate the structural model for NaChBac. It is, however, an exception to the “2–6 S4 cationic sidechain motif” and, as such, may be responsible, along with coupled pore structure differences, for the remarkably slower kinetics exhibited by this channel [57], [58].

### Local Network Interactions Sites in Na_v_ Channels. Functional Studies of Na_v_1.4

The replacement of native Asn by Cys in the **S2** segment of **D4** (N1392) of Na_v_1.4 is associated with a major perturbation of channel energetics. This result is strong experimental evidence that something similar to the postulated interactions in Shaker between **S4** and other transmembrane segments also occurs in sodium channels (see below).

Although there is conservation of sidechains in **S2** segments of Na_v_1.4, K^+^ channels and NaChBac, there is greater similarity of **D1** with **D3** and **D2** with **D4** of Na_v_1.4 (**D2**N608 and **D4**N1382) leading to the speculation that: first, specialization of domains may be present in Na^+^ channels related to functional differences (such as the greater association of **D4** with inactivation); second, that **D1** and **D3** of Na^+^ channels may have been constrained more evolutionarily than **D2** and **D4** from a common precursor ion channel; third, that a second duplication of a primordial channel containing two domains, having already previously duplicated in order to fashion a two-domain “evolutionary intermediate” (itself derived from duplication of a single-domain precursor), likely occurred; and, fourth, that polar but non-charged residues can replace anionic residues (*i.e.*, N608 and N1382 for glutamate) without loss of function in naturally occurring channels.

With the occurrence in Na_v_1.4 of neutral Asn at what would be the outermost anionic site of S2 (**D2**N608 and **D4**N1382) what becomes of the electrostatic interactions? One possibility is that the Asn is involved in interactions other than electrostatic (*e.g.*, H-bonding) but membrane helical interfacial pairwise contact propensities provide little support for a postulated Asn-Arg interaction [59].

The fact that cysteine substitution at N1392 does not abrogate function raises the question of whether it might be anionic. The value of the cysteine pK_a_ in proteins depends upon: (i) proximity of charged residues, (ii) dipole character of the α-helix, and (iii) hydrogen bonding to other residues none of which is known yet for the Na^+^ channel inactivated state. Therefore, the fact that cysteine substitutions do not eliminate channel function is not readily interpretable since the sidechain might be neutral, if the pK_a_ value is little perturbed (nominal pK_a_ ∼8.5–9), or might be substantially negative, if the pK_a_ is shifted to ∼7.5 (aided by a nearby **S4**Arg) [60]. However, the reaction with MTSET, placing a positive charge and a bulkier sidechain at this position, also does not completely interfere with channel function indicating that at least in the context of a single domain alteration, this site is reasonably permissive to change (Appendix S1, Tables A and B).

Experiments with N1382C without and with MTSET reaction reveal only small perturbations of activation perhaps because **D4** is more heavily involved in inactivation [3], [40]. Consistent with this concept there are larger effects upon inactivation involving depolarizing shifts in h_∞_-V midpoints with increased values of τ_h_ and decreased values of τ_rec_.

Most of the S2 mutations were associated with rightward shifts in h_∞_ midpoints as a result of increases in the rate constant for exit from (α) and decreases in the rate constant for entry into (β) the inactivated state. Although the nature of the changes in conformation of the V-domain of Na_v_1.4 are not yet known in detail, the increased instability of the inactivated state relative to non-inactivated states due to these mutations suggests that the residue substitutions interfere with favorable interactions in the inactivated state to a greater extent than they do in the non-inactivated states (primarily open states). The energetic perturbations are similar quantitatively for the N1382C and E1392C mutations despite the fact that there may be no change in charge in the case of 1382 (Asn → Cys) while the charge at 1392 is reduced from −1 to 0 (Glu → Cys). Rationalization of this in terms of electrostatic networks will require further experimentation.

The observed shifts in h_∞_-V midpoints (Table 2) are the first experimental evidence for sodium channels that **D4** is capable of network interactions involving **S2** (N1382) [32] (*c.f.*, [61]). It remains to be seen whether the same is true for other domains and to what extent they contribute to functional differences between K^+^ and Na^+^ channels [62], [63], [64], [65], [66], [67], [68], [69].

### SCAM

Only residues at the first N-terminal α-helical turn appear to be reactive to MTS reagents (1378, 1382) suggesting that aqueous crevices are either not present, quite shallow or blocked (Appendix S1, Tables A and B) (*c.f.*, [70]). This is consistent with recent data on Shaker in which S2 segments are solvent inaccessible and probably not associated with aqueous crevices [71]. There is no state-dependence of MTS reactivity consistent with related observations on K^+^ channels [49] (*c.f.*, [24]).

## Materials and Methods

### Mutagenesis

Site-directed substitutions were carried out using the Altered sites II *in vitro* mutagenesis system according to the manufacturer's instructions (Promega Corp., Madison WI) and synthetic antisense mutagenic oligonucleotide containing desired mutations with the introduction or removal of a restriction enzyme site to aid screening. All mutations were confirmed by dideoxynucleotide sequence determination.

### Transfection of tsA201 Cell Line

The tsA20l cell line, a mammalian cell line derived from human embryonic kidney HEK 293 cells by stable transformation with simian virus 40(SV40) T-antigen [72], was grown in high glucose DMEM, supplemented with 10% fetal bovine-serum, 2 mM L-glutamine, penicillin (100 U/ml) and streptomycin (10 mg/mI) (Gibco-BRL-Invitrogen, Carlsbad CA), in 5% CO_2_ humid atmosphere incubator. The tsA201 cells were transfected with WT or mutant rNa_v_1.4 cDNA contained in the mutagenesis and expression vector pAlter-Max (Promega) (10 ug) [73] along with human β_1_-subunit (10 ug) cDNA utilizing the calcium phosphate method and grown to 40–50% confluence on 100 mm plates [72], [74]. To facilitate the identification of individual transfected cells, 10 ug pHOOK-1, a plasmid expressing a single-chain antibody (sFv) directed against 4-ethoxymethylene-2-phenyl-2-oxazolin-5-one (phOx) on the surface of transfected cells, was also cotransfected simultaneously. For patch-clamp experiments, the cells were studied generally 2 to 3 days post transfection. Cells expressing sFv on their surface become decorated with Capture-Tec™ Beads (Invitrogen), which are magnetic beads coated with phOx after 30 min incubation when used according to the directions from the manufacturer and, thus, are easily distinguishable from untransfected cells. Plasmid DNA was purified using Qiagen Maxi-kits, (Qiagen Inc., Valencia CA). All of the mutants expressed Na^+^ selective currents.

### Patch-Clamp Methods

Macroscopic sodium currents from transfected cells were recorded using the whole-cell patch-clamp technique [75]. Patch electrodes were made from 8161 Corning borosilicate glass and coated with Sylgard® (Dow-Corning Corp., Midland MI) to minimize their capacitance. Voltage clamp was accomplished using low resistance-electrodes (∼2 MΩ) with leak subtraction (P/4) and series resistance compensation applied routinely, the latter to values >85%, to minimize voltage-clamp errors using an Axopatch 200B patch-clamp amplifier (Axon Instruments Inc., Union City CA). Voltage-clamp command pulses were generated by microcomputer using pCLAMP 6 software (Axon Instruments Inc., Union City CA). Recorded membrane currents were low-pass filtered at 5 kHz, digitized at 10 kHz, and stored on a PC equipped with and analog-to-digital converter (Digidata 1200, Axon Instruments). For whole-cell recording, the patch pipette contained (mM): 35 NaCl 105 CsF 10, EGTA: and 10 Na-HEPES (pH 7.4). The bath solution contained (mM): 150 NaCl 2 KCl 1.5 CaCl_2_: 1. MgCl_2_ 10 glucose and 10 Na-HEPES (pH 7.4). Experiments were performed at room temperature, 20–23°C. To minimize the influence of time-dependent shifts in the steady-state inactivation curves and systematic errors, recordings for rNa_v_1.4 were routinely made in the same order: I–V curve, steady-state inactivation, recovery from inactivation (HP = −120 mV) starting ten min after breaking the membrane. The cycle time between voltage steps was 2 sec.

### Analysis of Perturbations of Steady-State Parameters

We employed a two state model (single transitions between either non-open and open or non-inactivated and inactivated states) to evaluate the gating properties of the channel. Activation and inactivation curves were fit using a Boltzmann function *I/I_max_ = 1/(1 + exp[−zF(V−V_0.5_)/RT])* , where *V_0.5_* is the midpoint potential for activation or inactivation, *F* is the Faraday constant, *z* is the equivalent charge, *R* is the gas constant and *T* is temperature in ^o^ Kelvin. The ΔG_o_ values for activation or inactivation represent the free energy of channel opening or inactivation at zero voltage and measure the intrinsic stability of the open or inactivated conformation with respect to non-open or non-inactivated states, where ΔG_o_ = z*F V_0.5_*. The perturbation of the free energy for any given amino acid is the difference between the value for the substituted channel (MUT) and that of the native channel (WT): ΔΔG_o_ *= *ΔG_o_^MUT^−ΔG_o_^WT^ for each residue.

### Voltage-Dependence of Time constants of Current Decay and Recovery from Fast Inactivation

Time constants (τ_h_) for current decay were obtained by fits to single exponentials of the current traces. Time constants (τ_rec_) for recovery from fast inactivation were obtained by a single exponential fit to the time course of recovery in a double pulse protocol in which inactivation is induced by a 40 ms prepulse to −20 mV, followed by a variable duration recovery at different conditioning potentials, and then tested by a depolarization to −20 mV. If channel entry and exit from inactivation conforms to a two-state model, the voltage-dependent first-order rate constants for leaving (α) and entering (β) an inactivated state determine the observed time constants for recovery from fast inactivation (τ_rec_) and the time constants for current decay (τ_h_): the observed time constants (τ_rec_ and τ_h_) should be equivalent at any voltage and will equal 1/(α+β). The rate constants α and β were assumed to have an exponential dependence on membrane potential. The mutants and WT time constants are fit reasonably well by this simple, two-state model, where 
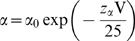
, and 
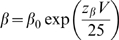
, *V* is the membrane potential in mV, 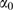 and 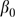 are the rate constants at 0 mV, 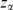 and 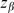 are equivalent charge in the gating process. The first-order rate constant for the channels entry into the inactivated state (β) at 0 mV is also given by transition state theory, 
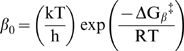
, and the first-order rate constant for the channel leaving the inactivated state at 0 mV (α) is 
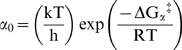
, where *k* is the Boltzmann constant, *h* is the Planck constant, *T* is the temperature in ^o^ Kelvin, *R* is the gas constant, 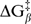 is the difference in the free energy between the open state and the transition state to the inactivated state at 0 mV, and 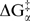 is the difference in the free energy between the inactivated state and the transition state to non-inactivated states at 0 mV. The perturbation of the free energy of entry into or exit from the inactivated state for any given amino acid is the difference between the value for the substituted channel and that of the native channel: 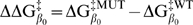 or 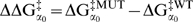 for each residue.

### Periodicity Analysis

A power spectrum Fourier transform analysis was carried out according to:
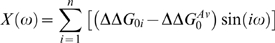
(1)

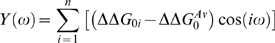
(2)

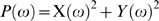
(3)where P(ω) is the Fourier transform power spectrum as a function of angular frequency, ω, ΔΔG_oi_ is the free energy value at a given position i, ΔΔG_o_ *^Av^*, is the average value of ΔΔG_oi_ for the segment, and n is the number of residues within the segment. ω is related to the number of residues/turn (d) where ω = 360/d. The ideal α-helical pattern is characterized by a maximum near ω = 360°/3.6 = 100° for 3.6 residues/turn. Helices within membranes tend to show peaks shifted to slightly higher frequencies [41]. All power spectra are shown without correction.



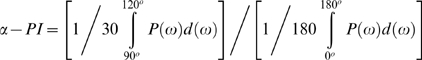
(4)


The number of residues on one side of a helix with similar perturbations can be characterized by the α-Periodicity Index (α-PI also called Ψ elsewhere), which is defined by the average value of P(ω) in the α-helical region 90°≤ω≤120° relative to the total average value of P(ω) [76]. α-PI can vary from 0 (no peak) to 6 (single peak for α-helical segment) although in practice, because of the imperfect nature of the analysis, noise, and deviations from α-helicity α-PI≥2 is taken to be indicative of a significant α-helical pattern.

### MTS Reactions

Because the reactivity of channel cysteine sidechains was generally greater with methanethiosulfonate-ethyltrimethlammonium ion (MTSET) than methanethiosulfonate-ethylsulfonate (MTSES), we concentrated on studies with the former compound. The stock solution 1.0 M of MTSET (Toronto Research Chemicals, Inc., Toronto, Canada) was prepared from the solid in DMSO (Sigma-Aldrich Corp., St. Louis MO), kept on the ice at the beginning of each half day, and diluted to a final concentration of 0.5 mM in the bath or the patch pipette solution immediately before use. MTSET solution was used within 10 min after dilution from the stock. The reagent (wash-in) or bath (wash-out) solution was perfused with a macropipette brought in apposition to the cell. In some experiments, the reagent was applied to a dish of cells for 10 min before washing the dish with the bath solution. For internal application, the reagent solution was placed in the patch pipette at the above concentrations and the tip back-filled with pipette solution devoid of reagent. Effects of sidechain reactions on electrophysiological characteristics of the modified channels were determined as described above for unreacted channels. Statistical significance was determined using a paired *Student's t test* with *P*<0.05 accepted as meaningful.

## Supporting Information

Appendix S1 (0.15 MB DOC)
