# Supplementary material for: Studies of α-Helicity and Intersegmental Interactions in Voltage-Gated Na+ Channels: S2D4
Source: PLoS One. 2009 Nov 2;4(11):e7674. doi: 10.1371/journal.pone.0007674 (PMC2766034; doi:10.1371/journal.pone.0007674)
Supplement: Appendix S1 — (0.15 MB DOC) [file pone.0007674.s001.doc]

**Appendix S1:**

**Table A. Gating Properties (G-V) of WT and S2D4 or S2-S3D4 Segment Mutants of Nav1.4 z (e0) (kcal/mol) (kcal/mol)**

| Channel | (mV) | z (e_0_) | (kcal/mol) | (kcal/mol) |
| --- | --- | --- | --- | --- |
| WT (11) | -31.4±1.04 | 4.04±0.10 | -2.94±0.17 |  |
| I1377C (9) | -32.0±1.19 | 4.32±0.16 | -3.21±0.22 | -0.27±0.24 |
| L1378C (10) | -32.8±1.08 | 4.25±0.11 | -3.22±0.17 | -0.28±0.15 |
| Y1379C (10) | -32.2±1.60 | 4.13±0.17 | -3.09±0.26 | -0.14±0.28 |
| N1380C (8) | -32.0±1.03 | 4.25±0.12 | -3.13±0.13 | -0.18±0.17 |
| I1381C (9) | -33.6±1.76 | 3.91±0.20 | -3.06±0.31 | -0.11±0.33 |
| N1382C (5) | -30.5±1.17 | 4.36±0.17 | -3.10±0.19 | -0.15±0.21 |
| M1383C (7) | -30.9±1.03 | 4.10±0.11 | -2.93±0.19 | 0.01±0.21 |
| V1384C (4) | -34.1±1.39 | 4.00±0.20 | -3.19±0.25 | -0.24±0.30 |
| F1385C (6) | -33.2±2.24 | 3.84±0.17 | -2.93±0.31 | -0.02±0.33 |
| I1386C (4) | -33.9±1.11 | 4.26±0.15 | -3.34±0.21 | -0.39±0.27 |
| I1387C (6) | -30.6±0.94 | 4.27±0.14 | -3.03±0.18 | -0.08±0.24 |
| I1388C (5) | -31.8±1.71 | 4.05±0.19 | -2.98±0.25 | -0.03±0.27 |
| F1389C (4) | -35.4±1.32 | 4.22±0.29 | -3.45±0.32 | -0.51±0.35 |
| T1390C (4) | -29.7±1.72 | 4.17±0.29 | -2.89±0.30 | -0.05±0.32 |
| G1391C (5) | -32.6±1.44 | 4.37±0.19 | -3.31±0.28 | -0.36±0.31 |
| E1392C (15) | -29.6±1.21 | 4.35±0.19 | -2.99±0.23 | -0.05±0.26 |
| C1393Q (5) | -30.9±1.55 | 4.11±0.16 | -2.92±0.16 | 0.02±0.23 |
| C1393W (4) | -30.0±1.70 | 3.69±0.18 | -2.58±0.24 | 0.36±0.27 |
| V1394C (10) | -32.2±1.07 | 4.30±0.08 | -3.19±0.09 | -0.25±0.21 |
| L1395C (6) | -31.2±1.13 | 4.26±0.14 | -3.09±0.21 | -0.15±0.24 |
| K1396C (15) | -29.7±1.92 | 4.16±0.31 | -2.89±0.35 | -0.06±0.39 |
| M1397C (11) | -31.8±1.03 | 4.31±0.12 | -3.09±0.17 | -0.15±0.23 |
| F1398C (7) | -32.1±0.82 | 4.07±0.16 | -3.01±0.16 | -0.07±0.23 |
| A1399C (4) | -34.0±2.80 | 3.91±0.20 | -3.08±0.39 | -0.14±0.42 |
| R1401C (5) | -30.26±1.05 | 4.11±0.21 | -2.87±0.18 | 0.07±0.25 |
| F1405C (7) | -31.8±0.63 | 3.97±0.13 | -2.91±0.12 | 0.03±0.20 |
| W1409C (5) | -32.5±1.17 | 4.10±0.13 | -3.07±0.13 | -0.13±0.21 |
| L1378C (4) ^‡^ | -29.9±1.84 | 4.44±0.13 | -3.08±0.27 | -0.14±0.31 |
| N1382C (7) ^‡^ | -33.7±1.15 | 4.76±0.13 | -3.78±0.21 | -0.62±0.29 |

The first column shows the residue number, identity in WT channel, the nature of the mutation, and, in parentheses, the number of cells. Native cysteine was mutated to tryptophan and glutamine. *V_0.5_* (midpoint potential) and z (equivalent charge) were obtained from fits to single Boltzmann equations (Fig. 1). The free energy difference at 0 mV between closed and open states () was calculated as described in Materials and Methods. The difference between WT and mutant values is . ^‡^MTSET (0.5 mM) is applied extracellularly. Data are mean values ± SEM.

**Appendix S1:**

**Table B. Gating Properties (h∞-V) of WT and S2D4 or S2-S3D4 Segment Mutants of Nav**1.4

| Channel | | (mV) | z (e_0_) | (kcal/mol) | | (kcal/mol) | |  | | |  | |
| --- | --- | --- | --- | --- | --- | --- | --- | --- | --- | --- | --- | --- |
|  | WT (11) | -72.2±0.58 | 5.85±0.10 | | -9.77±0.18 | |  | | 0.41±0.01 | 9.52±0.47 | |  |
|  | I1377C (9) | -71.0±0.89 | 5.69±0.11 | | -9.32±0.17 | | 0.44±0.27 | | 0.40±0.06 | 9.67±0.57 | |  |
|  | L1378C (10) | -71.7±0.66 | 5.57±0.18 | | -9.21±0.33 | | 0.57±0.35 | | 0.43±0.05 | 10.8±0.68 | |  |
|  | Y1379C (10) | -70.1±0.92* | 5.53±0.12* | | -8.93±0.24* | | 0.84±0.29 | | 0.36±0.01* | 7.07±0.37* | |  |
|  | N1380C (8) | -70.8±0.86 | 5.86±0.10 | | -9.57±0.11 | | 0.21±0.20 | | 0.38±0.01 | 10.6±0.98 | |  |
|  | I1381C (9) | -73.6±0.53 | 5.66±0.14 | | -9.59±0.21 | | 0.18±0.27 | | 0.44±0.04 | 11.3±0.64 | |  |
|  | N1382C (5) | -62.4±1.09** | 4.58±0.34** | | -6.74±0.42** | | 3.03±0.46 | | 0.64±0.07* | 4.96±0.08** | |  |
|  | M1383C (7) | -70.2±1.13 | 5.79±0.16 | | -9.37±0.25 | | 0.40±0.31 | | 0.39±0.01 | 9.02±0.88 | |  |
|  | V1384C (4) | -73.2±1.15 | 5.84±0.09 | | -9.85±0.12 | | -0.08±0.21 | | 0.40±0.05 | 9.74±0.46 | |  |
|  | F1385C (6) | -78.2±0.92** | 5.55±0.12 | | -10.01±0.20 | | -0.23±0.27 | | 0.28±0.04* | 12.2±0.71* | |  |
|  | I1386C (4) | -65.9±0.57** | 5.27±0.05** | | -8.01±0.07** | | 1.77±0.19 | | 0.67±0.03** | 2.67±0.43** | |  |
|  | I1387C (6) | -71.8±1.35 | 5.91±0.15 | | -9.77±0.14 | | 0.01±0.22 | | 0.36±0.02 | 10.5±0.55 | |  |
|  | I1388C (5) | -70.6±1.65 | 5.75±0.09 | | -9.35±0.09 | | 0.42±0.20 | | 0.40±0.01 | 8.74±1.19 | |  |
|  | F1389C (4) | -69.3±0.90* | 4.79±0.18** | | -7.67±0.30** | | 2.11±0.34 | | 0.63±0.04** | 5.48±0.74** | |  |
|  | T1390C (4) | -63.1±1.04** | 5.86±0.11 | | -8.54±0.36* | | 1.23±0.39 | | 0.45±0.02* | 4.51±0.27** | |  |
|  | G1391C (5) | -71.6±1.11 | 5.72±0.10 | | -9.45±0.18 | | 0.32±0.25 | | 0.39±0.12 | 8.36±1.09 | |  |
|  | E1392C (7) | -62.0±1.05** | 4.65±0.14** | | -6.70±0.28** | | 3.09±0.33 | | 1.24±0.09** | 3.21±0.13** | |  |
|  | C1393Q (5) | -70.1±1.09 | 5.67±0.29 | | -9.13±0.33 | | 0.64±0.37 | | 0.55±0.05 | 10.7±1.15 | |  |
|  | C1393W (4) | -74.0±1.21 | 5.39±0.18 | | -9.25±0.49 | | 0.52±0.27 | | 0.47±0.05 | 12.1±2.04 | |  |
|  | V1394C (10) | -71.8±0.79 | 5.88±0.05 | | -9.73±0.03 | | 0.05±0.18 | | 0.41±0.01 | 9.91±1.65 | |  |
|  | L1395C (6) | -70.7±0.81 | 5.99±0.16 | | -9.79±0.26 | | -0.02±0.31 | | 0.35±0.02 | 9.98±0.07 | |  |
|  | K1396C (15) | -64.4±0.62** | 5.59±0.08* | | -8.33±0.16** | | 1.16±0.23 | | 0.57±0.02** | 3.42±0.33** | |  |
|  | M1397C (11) | -72.0±0.73 | 5.93±0.14 | | -9.82±0.15 | | -0.04±0.34 | | 0.37±0.02 | 11.6±2.18 | |  |
|  | F1398C (7) | -73.0±0.87 | 5.91±0.11 | | -9.98±0.29 | | -0.21±0.11 | | 0.35±0.02 | 11.6±1.53 | |  |
|  | A1399C (4) | -73.3±1.12 | 5.66±0.13 | | -9.58±0.39 | | 0.19±0.18 | | 0.38±0.05 | 10.7±1.44 | |  |
|  | L1400C (12) | -72.2±0.81 | 5.69±0.09 | | -9.50±0.15 | | 0.27±0.23 | | 0.48±0.05 | 11.6±0.48 | |  |
|  | R1401C (5) | -70.0±1.12 | 5.89±0.15 | | -9.54±0.35 | | 0.23±0.39 | | 0.40±0.04 | 9.78±1.08 | |  |
|  | F1405C (7) | -71.5±0.33 | 5.64±0.08 | | -9.28±0.10 | | 0.42±0.19 | | 0.37±0.04 | 10.6±0.68 | |  |
|  | W1409C (5) | -71.9±0.48 | 5.72±0.18 | | -9.47±0.27 | | 0.29±0.32 | | 0.39±0.06 | 9.61±0.98 | |  |
|  | L1378C (4)^‡^ | -58.2±1.20** | 4.63±0.25** | | -6.23±0.46** | | 2.97±0.57 | | 1.27±0.05** | 2.60±1.21** | |  |
|  | N1382C (7)^‡^ | -56.0±0.81** | 4.03±0.16* | | -5.20±0.11** | | 1.54±0.44 | | 1.01±0.05** | 2.94±0.11* | |  |

The first column shows the residue number, identity in WT channel, the nature of the mutation, and, in parentheses, the number of cells. Native cysteine was mutated to tryptophan and glutamine. *V_0.5_* (midpoint potential) and z (equivalent charge) were obtained from fits to single Boltzmann equations (Fig. 2). The free energy difference at 0 mV between closed and inactivated states (ΔG_o_) was calculated as described in Materials and Methods. The difference between WT and mutant values is ΔΔG_o_. The current decay time constants (τ_h_) were obtained by fitting the current decays to a single exponential for each voltage and the voltage-dependence was determined by fitting to a voltage-dependent exponential (Materials and Methods): τ_h_^o^ is the τ_h_ value at 0 mV. τ_rec_^-90^ is the time constant for recovery from the fast inactivation at -90 mV. ^‡^MTSET (0.5 mM) is applied extracellularly. Data are mean values ± SEM. * represents student’s t-test is significant compared to WT (P<0.05), ** P<0.005. Data are mean values ± SEM.

**Appendix S**

**Table C. The Rate Constants of WT and S2D4 Segment Mutants of rNav1.4 for Entry Into and Exit From the Fast Inactivated State.**

|  | | (1/ms) | | $(e_{0})$ | | (1/ms) | | | $(e_{0})$ | ΔG_α0_^‡^ (kcal/mol) | | | ΔG_β0_^‡^. (kcal/mol) | ΔΔG_α0_^‡^ (kcal/mol) | | | ΔΔG_β0_^‡^. (kcal/mol) | |
| --- | --- | --- | --- | --- | --- | --- | --- | --- | --- | --- | --- | --- | --- | --- | --- | --- | --- | --- |
|  | WT (11) | | 3.5 | | 2.8 | | 10 | 2.25 | | | 20.93 | 12.07 | | |  |  | |  |
|  | I1377C (9) | | 3.65 | | 2.8 | | 10.5 | 2.4 | | | 20.9 | 12.04 | | | -0.03 | -0.03 | |  |
|  | L1378C (10) | | 3.45 | | 2.8 | | 10.4 | 2.3 | | | 20.93 | 12.04 | | | 0.01 | -0.02 | |  |
|  | Y1379C(10) | | 7.5 | | 2.85 | | 5 | 2.45 | | | 20.47 | 12.48 | | | -0.45 | 0.41 | |  |
|  | N1380C (8) | | 3.55 | | 2.75 | | 10.5 | 2.45 | | | 20.92 | 12.04 | | | -0.01 | -0.03 | |  |
|  | I1381C (9) | | 3.6 | | 2.75 | | 9.5 | 2.25 | | | 20.91 | 12.1 | | | -0.02 | 0.03 | |  |
|  | N1382C (5) | | 25 | | 2.45 | | 3.5 | 2.35 | | | 19.75 | 12.69 | | | -1.17 | 0.63 | |  |
|  | M1383C (7) | | 3.55 | | 2.8 | | 11.5 | 2.35 | | | 20.92 | 11.99 | | | -0.01 | -0.08 | |  |
|  | V1384C (4) | | 3.3 | | 2.75 | | 10.5 | 2.35 | | | 20.96 | 12.04 | | | 0.04 | -0.03 | |  |
|  | F1385C (6) | | 2 | | 2.8 | | 12.5 | 2.25 | | | 20.26 | 11.94 | | | 0.33 | -0.13 | |  |
|  | I1386C (4) | | 10 | | 2.85 | | 3.5 | 2.25 | | | 20.3 | 12.69 | | | -0.63 | 0.63 | |  |
|  | I1387C (6) | | 3.65 | | 2.75 | | 11 | 2.35 | | | 20.9 | 12.01 | | | -0.03 | -0.06 | |  |
|  | I1388C (5) | | 3.55 | | 2.75 | | 9.5 | 2.25 | | | 20.92 | 12.1 | | | -0.01 | 0.03 | |  |
|  | F1389C (4) | | 7 | | 2.7 | | 4.5 | 2.25 | | | 20.51 | 12.54 | | | -0.41 | 0.48 | |  |
|  | T1390C (4) | | 7.5 | | 2.8 | | 5.5 | 2.25 | | | 20.47 | 12.42 | | | -0.45 | 0.36 | |  |
|  | G1391C (5) | | 3.5 | | 2.8 | | 10 | 2.25 | | | 20.93 | 12.07 | | | 0 | 0 | |  |
|  | E1392C (15) | | 20 | | 2.4 | | 2 | 2.25 | | | 19.75 | 12.79 | | | -1.17 | 0.72 | |  |
|  | C1393 Q(5) | | 3.65 | | 2.65 | | 9.5 | 2.65 | | | 20.9 | 12.1 | | | -0.03 | 0.03 | |  |
|  | C1393 W(4) | | 3.55 | | 2.65 | | 9.5 | 2.25 | | | 20.92 | 12.1 | | | -0.01 | 0.03 | |  |
|  | V1394C(10) | | 3.4 | | 2.7 | | 9.5 | 2.35 | | | 20.94 | 12.1 | | | 0.02 | 0.03 | |  |
|  | L1395C (6) | | 3.7 | | 2.7 | | 9.8 | 2.45 | | | 20.89 | 12.08 | | | -0.03 | 0.01 | |  |
|  | K1396C(15) | | 15 | | 2.65 | | 4.5 | 2.15 | | | 20.06 | 12.54 | | | -0.87 | 0.48 | |  |
|  | M1397C(11) | | 3.45 | | 2.65 | | 9.6 | 2.45 | | | 20.93 | 12.09 | | | 0.01 | 0.02 | |  |
|  | F1398C (7) | | 3.7 | | 2.65 | | 11 | 2.45 | | | 20.89 | 12.01 | | | -0.03 | -0.06 | |  |
|  | A1399C (4) | | 3.55 | | 2.75 | | 10.8 | 2.35 | | | 20.92 | 12.02 | | | -0.01 | -0.05 | |  |
|  | L1400 (12) | | 3.5 | | 2.75 | | 10.5 | 2.35 | | | 20.93 | 12.04 | | | 0.01 | -0.03 | |  |

The first column shows the residue number, identity in WT channel, the nature of the mutation, and, in parentheses, the number of cells. Native cysteine was mutated to glutamine and tryptophan. α0 and β0 represent the voltage-dependent rate constants for leaving and entering an inactivated state at 0 mV, respectively. *zα* and *zβ* were equivalent charge in the gating proceeds for leaving and entering an inactivated state, respectively. ΔΔG_β0_^‡^ is the difference in the free energy between the transition state and the resting/close state at 0 mV, and ΔΔG_α0_^‡^ is the difference in the free energy between the transition state and the inactivated state at 0 mV, which were calculated as described in Materials and Methods. The differences between WT and mutant values are ΔΔG_α0_^‡^ and ΔΔG_β0_^‡^. Data are mean values ± SEM.
